# Supplementary figures and images for: An oomycete effector subverts host vesicle trafficking to channel starvation-induced autophagy to the pathogen interface
Source: eLife. 2021 Aug 23;10:e65285. doi: 10.7554/eLife.65285 (PMC8382295; doi:10.7554/eLife.65285)

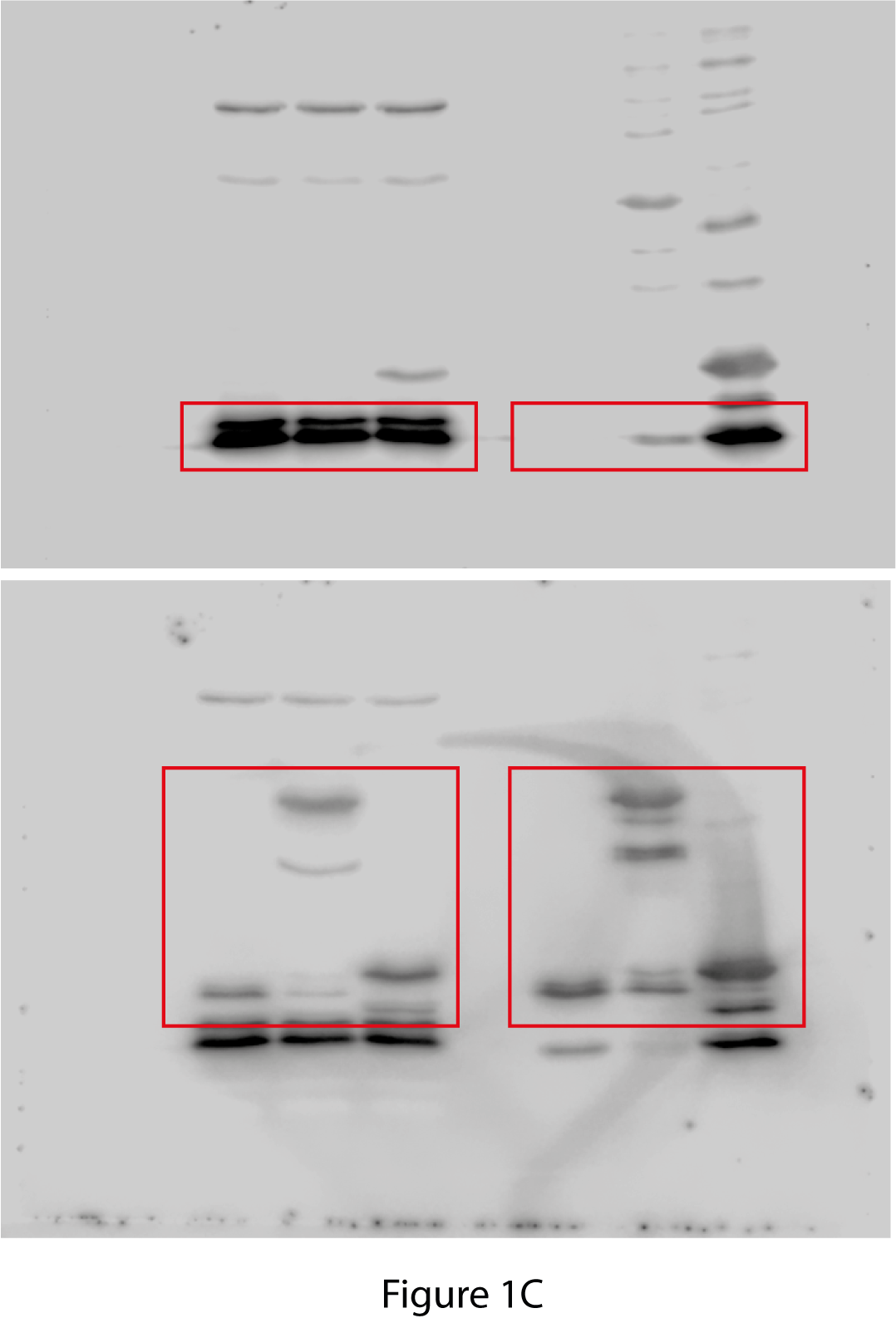

Supplement: Figure 1—source data 1. — The ZIP archive contains full sized immune blots and data sheet values for autophagosome quantification. [file elife-65285-fig1-data1.zip › Figure 1/Figure 1 WBs.tif]

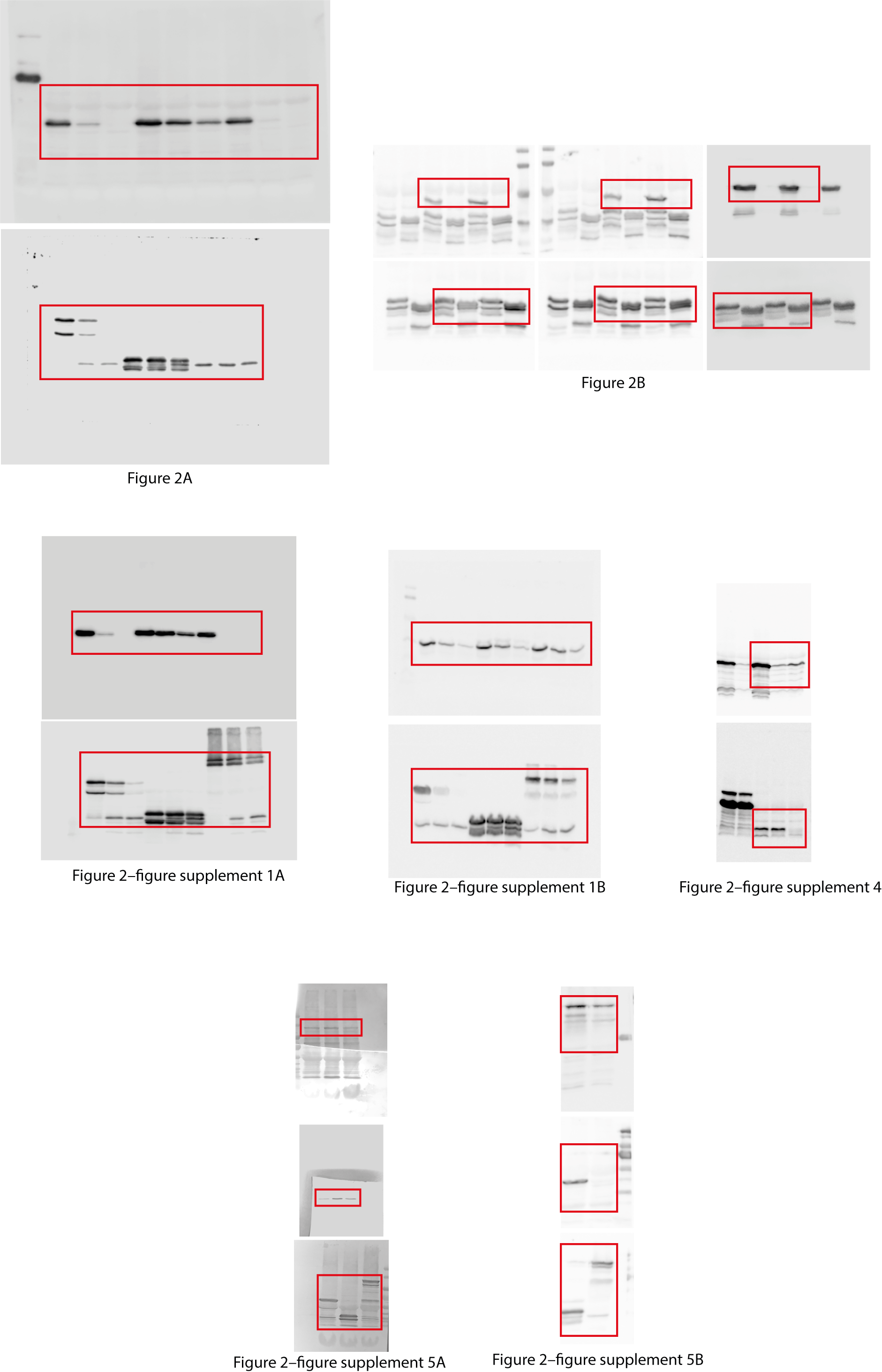

Supplement: Figure 2—source data 1. — The ZIP archive contains full sized immune blots and data sheet values for autophagosome quantification. [file elife-65285-fig2-data1.zip › Figure 2-source data 1/Figure 2 WB.tif]

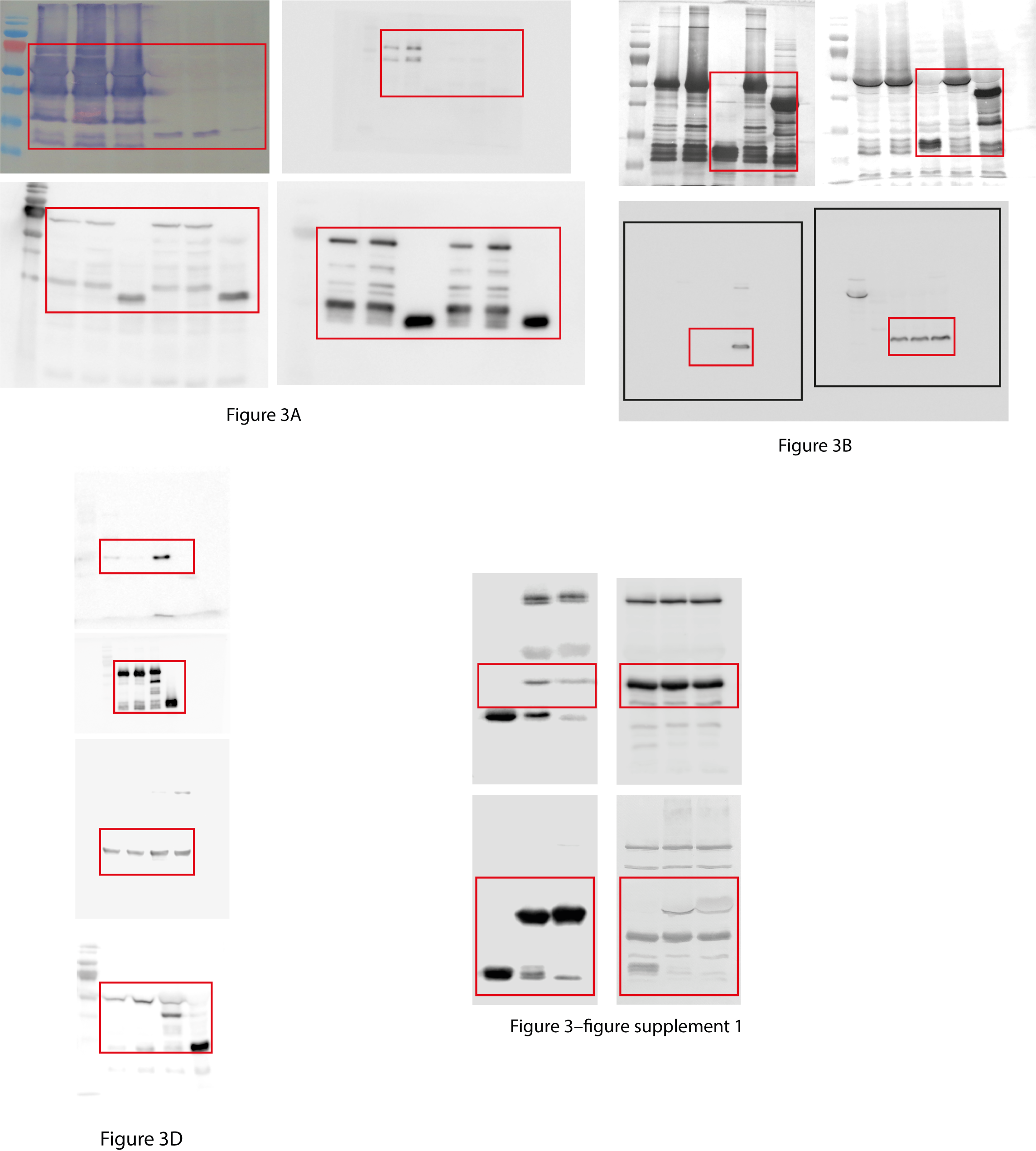

Supplement: Figure 3—source data 1. — The ZIP archive contains full sized immune blots, Rab8a wt and mutant sequences, and data sheet values for autophagosome quantification and colocalization assays. [file elife-65285-fig3-data1.zip › Figure 3-source data 1/Figure 3 WB.tif]

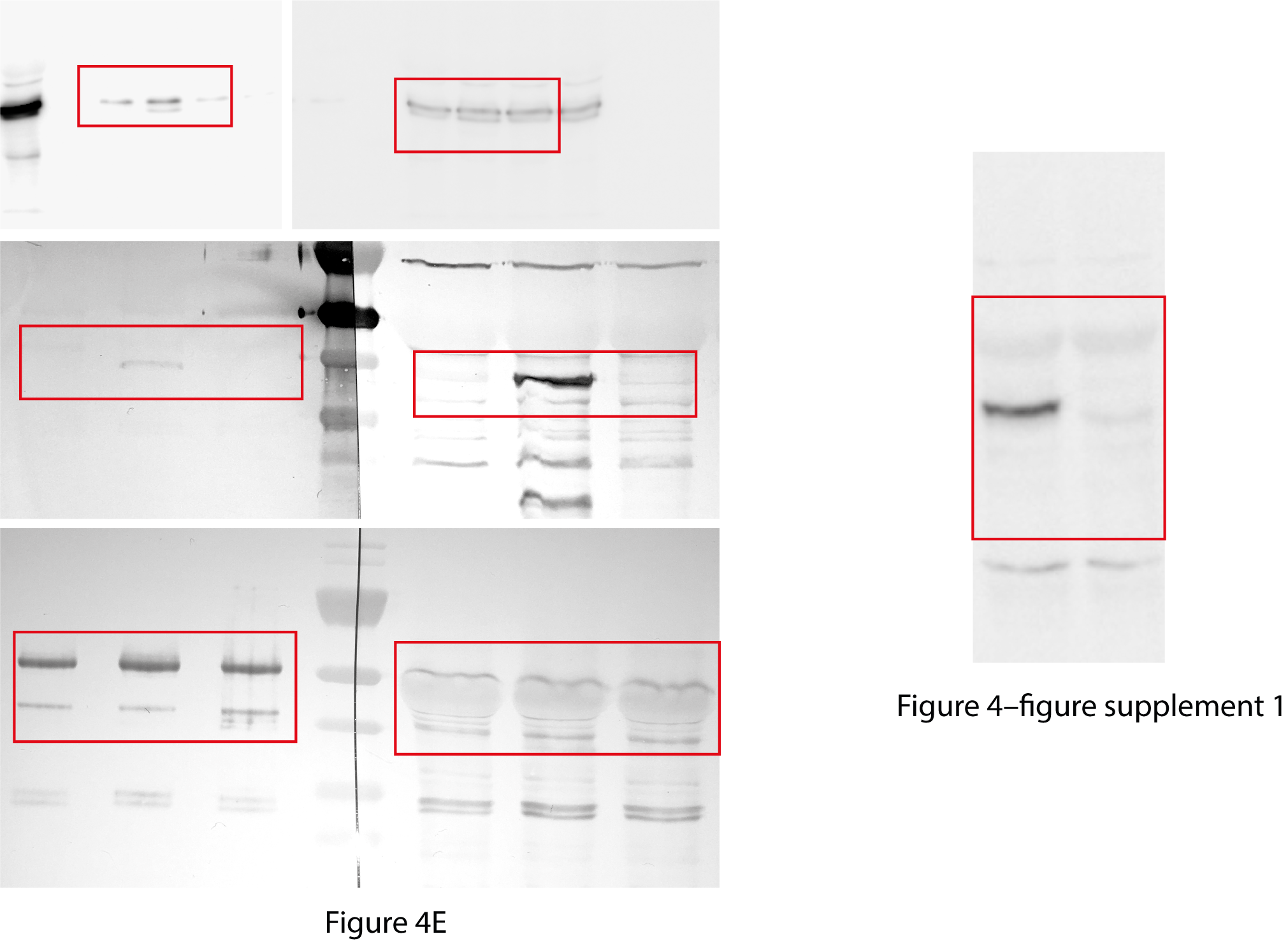

Supplement: Figure 4—source data 1. — The ZIP archive contains full sized immune blots, data sheet values for colocalization analyses, and Fiji Macro/plugin used for puncta colocalization. [file elife-65285-fig4-data1.zip › Figure 4-source data 1/Figure 4 WB.tif]

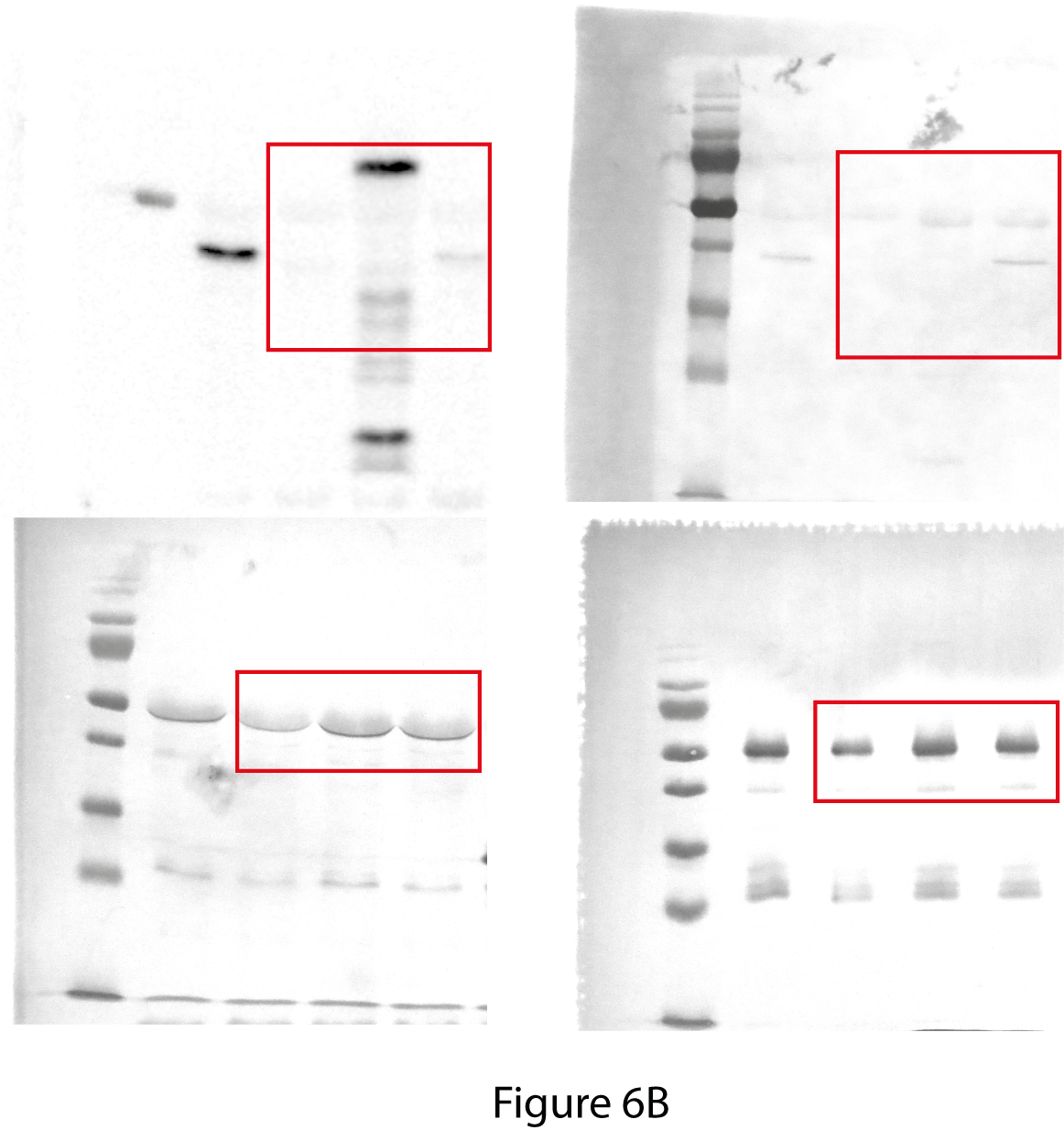

Supplement: Figure 6—source data 1. — The ZIP archive contains uncropped full sized immune blots, data sheet values for autophagosome quantification and colocalization assays. [file elife-65285-fig6-data1.zip › Figure 6-source data 1/Figure 6 WB.tif]

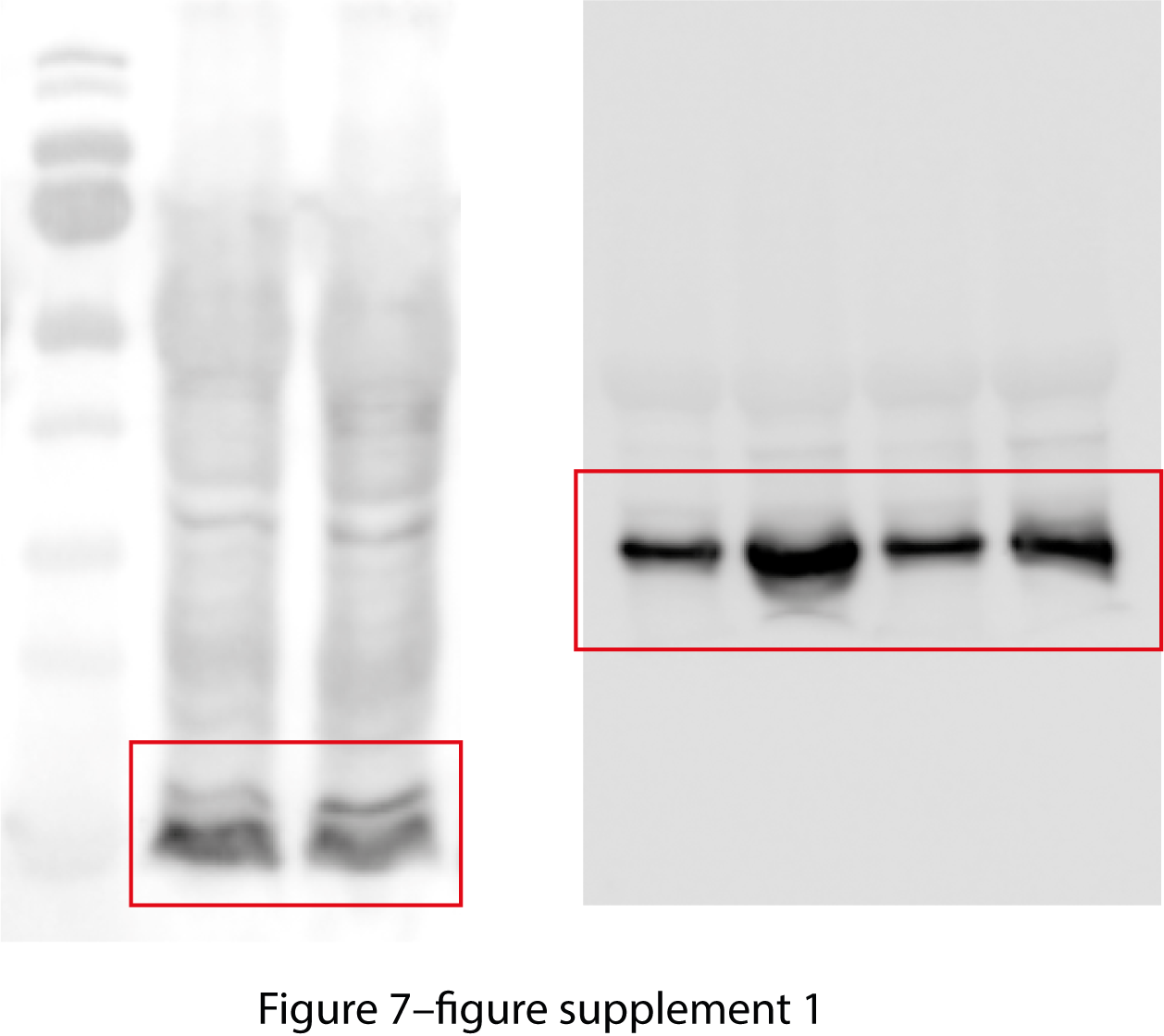

Supplement: Figure 7—source data 1. — The ZIP archive contains uncropped full sized immune blots, data sheet values for autophagosome quantification and colocalization assays. [file elife-65285-fig7-data1.zip › Figure 7-source data 1/Figure 7 WB.tif]
